# Constructive Neuroengineering of Crossing Multi-Neurite Wiring Using Modifiable Agarose Gel Platforms

**DOI:** 10.3390/gels11060419

**Published:** 2025-05-30

**Authors:** Soya Hagiwara, Kazuhiro Tsuneishi, Naoya Takada, Kenji Yasuda

**Affiliations:** 1 Department of Pure and Applied Physics, Graduate School of Advanced Science and Engineering, Waseda University, 3-4-1 Okubo, Shinjuku 169-8555, Tokyo, Japan; soyahagiwara@ruri.waseda.jp (S.H.); shikon.purple@fuji.waseda.jp (N.T.); 2 Department of Physics, School of Advanced Science and Engineering, Waseda University, 3-4-1 Okubo, Shinjuku 169-8555, Tokyo, Japan; kazu140613@akane.waseda.jp

**Keywords:** neuron, hippocampal cell, neurite elongation control, agarose gel micropattern, bending microchannel array, bending angle, neuronal network formation

## Abstract

Constructing stable and flexible neuronal networks with multi-neurite wiring is essential for the in vitro modeling of brain function, connectivity, and neuroplasticity. However, most existing neuroengineering platforms rely on static microfabrication techniques, which limit the ability to dynamically control circuit architecture during cultivation. In this study, we developed a modifiable agarose gel-based platform that enables real-time microstructure fabrication using an infrared (IR) laser system under live-cell conditions. This approach allows for the stepwise construction of directional neurite paths, including sequential microchannel formation, cell chamber fabrication, and controlled neurite–neurite crossings. To support long-term neuronal health and network integrity in agarose microstructures, we incorporated direct glial co-culture into the system. A comparative analysis showed that co-culture significantly enhanced neuronal adhesion, neurite outgrowth, and survival over several weeks. The feeder layer configuration provided localized trophic support while maintaining a clear separation between glial and neuronal populations. Dynamic wiring experiments further confirmed the platform’s precision and compatibility. Neurites extended through newly fabricated channels and crossed pre-existing neurites without morphological damage, even when laser fabrication occurred after initial outgrowth. Time-lapse imaging showed a temporary growth cone stalling at crossing points, followed by successful elongation in all tested samples. Furthermore, the direct laser irradiation of extending neurites during microstructure modification did not visibly impair neurite elongation, suggesting minimal morphological damage under the applied conditions. However, potential effects on molecular signaling and electrophysiological function remain to be evaluated in future studies. Together, these findings establish a powerful, flexible system for constructive neuroengineering. The platform supports long-term culture, real-time modification, and multidirectional wiring, offering new opportunities for studying neural development, synaptic integration, and regeneration in vitro.

## 1. Introduction

The ability to reconstruct and manipulate neuronal connectivity in vitro is fundamental to understanding how information is processed, transmitted, and stored in neural systems. Historically, axons were conceptualized as passive transmission lines, relaying stereotypical all-or-none action potentials (APs) from the soma to presynaptic terminals. However, emerging insights over the last decade reveal that axons exhibit rich and dynamic computational behaviors—including distal AP initiation, analog–digital synaptic modulation, and activity-dependent plasticity—that expand their functional roles in brain circuits [1,2,3,4].

These findings necessitate experimental models that enable the precise spatial control and dynamic modification of axonal trajectories. A wide range of in vitro axonal guidance strategies has been developed to manipulate growth cone behavior and streamline axon tracking. These techniques take advantage of the axon’s innate capacity for taxis—responding to environmental cues via motile growth cones. Historically, two major categories have emerged: physical patterning, which uses topographical and geometrical constraints, and chemical patterning, which employs molecular cues to attract or repel neurites [5,6,7,8].

Physical guidance strategies rely on micro- and nanofabricated substrates with defined surface features. Engineered grooves, ridges, gratings, or micropillars constrain neurite outgrowth direction and can influence branching and growth rate. For example, unidirectional linear grooves promote axon extension while reducing branching complexity [9], whereas discontinuous structures like pillar arrays can guide multiple branches along predefined tracks [10,11]. Substrate stiffness, geometry, and feature spacing can also impact neurite morphology and growth patterns [12]. Advanced approaches include microfluidic devices—such as those pioneered by Taylor et al.—that use long, narrow microchannels to compartmentalize axons from somatodendritic regions [13]. These designs have enabled high-resolution studies of axonal transport, injury response, and synaptic targeting.

Chemical patterning methods, on the other hand, use immobilized or soluble guidance cues. Microcontact printing (µCP) is widely employed to deposit adhesive molecules like laminin or poly-D-lysine in continuous or discontinuous patterns [14,15,16]. These patterns can be tuned to control axon elongation, branching, and even fasciculation. Hybrid approaches combine physical scaffolds with chemical gradients—for instance, using microfluidic devices to generate NGF gradients along physical channels [17].

While both strategies have the advanced in vitro modeling of axonal behavior, they suffer from a common limitation: most patterning is static. Substrates are fabricated before neuronal seeding, and once neurites begin to grow, the patterns cannot be changed or reconfigured. This rigid constraint precludes the real-time adaptation of network architectures and hinders investigations into developmental dynamics, injury-induced rewiring, or plasticity-driven circuit remodeling. Furthermore, prolonged cultures often face issues like neurite misrouting, overextension, or fasciculation beyond the intended path, particularly as networks mature [4,18].

To address these limitations, the field has moved toward Brain-on-Chip (BoC) biotechnology, which integrates microengineering, live-cell imaging, and electrophysiological tools to recapitulate brain-like microenvironments in vitro [13,14,15,19]. BoC platforms frequently rely on pre-patterned substrates and modular compartmentalization to mimic network motifs, but remain largely inflexible once established. Moreover, their integration with long-term culture systems, glial support, and high-throughput probing remains underdeveloped [20]. Recent innovations in in situ axon patterning—such as two-photon polymerization or photothermal melting—have created the possibility of modifying neural circuitry in real time [21,22,23,24].

Our group has previously developed a constructive agarose gel microfabrication system to address this challenge [22,24]. In our recent study, we demonstrated that steep-angle (≥120°) microchannel geometries fabricated in agarose gel could be used to precisely control neurite outgrowth length and prevent miswiring in open-ended neural circuits [18]. This method exploited the observation that neurite elongation is arrested at bending angles greater than a critical threshold (90°), providing a strategy for robust directional control without requiring enclosed barriers. By applying these steep-angle configurations, we enabled contact-based neurite junctions while eliminating unintended overextensions, thus laying the groundwork for single-neuron resolution wiring. However, while this approach successfully prevented misrouting, it remained geometrically static, limiting its utility for circuit adaptation or progressive wiring during cultivation.

To address these challenges, we present a real-time modifiable agarose gel platform for the constructive neuroengineering of the flexible wiring of neurites during cultivation for multi-neurite networks. Notably, this is the first demonstration of guided neurite crossing fabricated during active cell culture, which represents an essential step for enabling the stepwise and selective construction of functional neuronal networks. Using infrared (IR) laser-mediated melting, our system enables the on-demand fabrication of microchannels and cell chambers under live-culture conditions, preserving pre-existing neuronal viability and morphology. This approach is compatible with glial co-culture support, enhancing neuronal survival and long-term circuit stability. Importantly, it enables stepwise neurite wiring, controlled orthogonal crossing, and bidirectional axonal interactions—capabilities that are largely inaccessible with conventional patterning methods.

Our platform bridges the gap between classical neurobiological models and emerging BoC technologies. It offers a biocompatible, reconfigurable, and scalable system for investigating axonal function, synaptic specificity, and regenerative rewiring. By combining the principles of microsystems engineering, neurobiology, and tissue patterning, this work contributes to the next generation of brain-on-chip models—capable not only of structural mimicry, but also of adaptive circuit reconfiguration, a key feature of living neural systems.

## 2. Results and Discussion

### 2.1. Agarose Gel Neuronal Network Microfabrication System Design and Microfabrication Procedure

**Figure 1:**
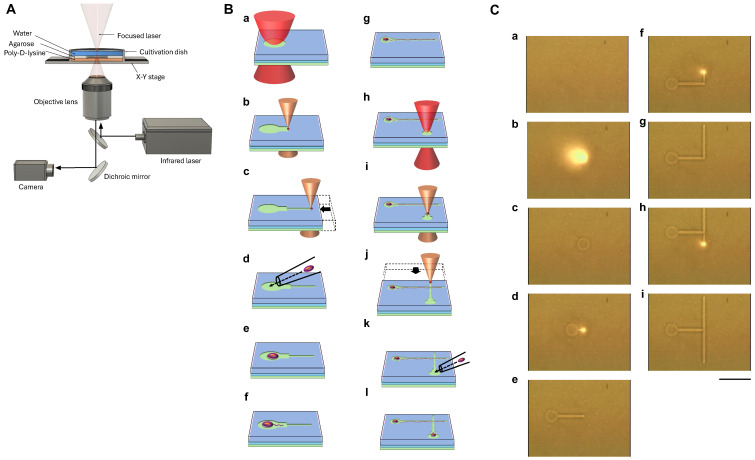
Experimental setup and agarose gel microfabrication procedure. (**A**) Schematic of the custom infrared (IR) laser-based system for agarose gel microstructure fabrication. The setup integrates a fiber-coupled IR laser, live imaging optics, and a motorized X–Y stage for real-time patterning under aqueous conditions. (**B**) Conceptual diagram of the fabrication sequence: (**a**) Localized laser melting creates the first cell chamber. (**b**,**c**) A microchannel is patterned by translating the stage. (**d**) A single neuron is manually placed in the chamber. (**e**–**g**) After several hours of culture, a neurite extends into the microchannel. (**h**–**j**) A second chamber is formed at the distal end. (**k**,**l**) A second neuron is placed, completing a directed two-cell circuit. This configuration enables neurites to cross orthogonally on a two-dimensional plane. (**C**) Sequential infrared micrographs of the laser fabrication process: (**a**) Agarose gel-coated dish prior to patterning. (**b**) Laser irradiation at 0.25 W for 2 s forms the first cell chamber, visualized by a bright laser spot. (**c**) A circular chamber appears post-irradiation. (**d**,**e**) A microchannel is fabricated by moving the stage (0.16 W, 5 μm/s). (**f**–**i**) Additional microchannels are created using the same process. The visible laser focus facilitates the precise alignment of new features with existing structures. Bright spots of infrared camera images represent the laser spot areas. Scale bar, 100 μm.

To enable precise control over neuronal network geometry, we developed a real-time, modifiable microfabrication system based on an infrared (IR) laser-mediated patterning of agarose gel substrates. As shown in Figure 1A, the system integrates a fiber-coupled IR laser, an inverted microscope with IR imaging capability, and a motorized X–Y stage. This configuration permits spatiotemporally controlled melting of the agarose gel layer under live-cell culture conditions without affecting cell viability.

The conceptual framework of the system is illustrated in Figure 1B. The fabrication sequence begins with the creation of a circular cell chamber by targeted IR laser irradiation (Figure 1(Ba)). The laser melts a localized region of agarose gel, removing the gel and exposing the underlying cultivation dish surface. A linear microchannel is then fabricated by translating the culture dish beneath the fixed laser beam, forming a defined path for neurite outgrowth (Figure 1(Bb,c)). A single dissociated neuron is introduced into the first chamber using a fine-tipped micropipette (Figure 1(Bd)). Following several hours of cultivation, a neurite extends into the fabricated microchannel, adopting a unidirectional trajectory (Figure 1(Be–g)). Once the desired elongation is achieved, a second cell chamber is generated at the distal end of the microchannel by additional laser melting (Figure 1(Bh–j)).This chamber serves as a placement site for a second neuron (Figure 1(Bk,l)),thus enabling the construction of a spatially constrained, two-node neuronal circuit with defined directional connectivity. In this example, neurites from different neurons can pass orthogonally through the pattern, allowing them to cross each other in a two-dimensional plane.

Figure 1C provides infrared micrographs capturing the actual fabrication process on the agarose gel layer. Prior to irradiation, a thin and uniform agarose gel layer covers the culture dish (Figure 1(Ca)). Upon the activation of the laser at 0.25 W for 2 s, a visible laser spot appears at the target site (Figure 1(Cb)). The melting of the agarose gel results in the formation of a distinct, circular cell chamber (Figure 1(Cc)). Continuous laser irradiation during stage translation at 5 μm/s (0.16 W) generates a narrow microchannel connected to the first chamber (Figure 1(Cd,e)). Additional microchannels are fabricated in a similar manner (Figure 1(Cf–i)), highlighting the modularity and scalability of the method. As shown in the micrographs, the ability to visualize the laser spot in real-time facilitates precise alignment during patterning, ensuring accurate connectivity between chambers.

To optimize agarose microstructure fabrication while minimizing thermal stress on surrounding cells, we carefully adjusted the infrared laser power during patterning. When focused, the 1480 nm laser locally heats the agarose layer, causing solation. The resulting increase in temperature induces the immediate convection of the surrounding medium, which rapidly diffuses both heat and the solated agarose material away from the irradiation site. This convective flow enables the irradiated region to quickly reach thermal equilibrium. During calibration, we gradually increased the laser intensity and determined the minimal power required to achieve consistent agarose melting. By operating at this threshold level, the temperature at the laser focus remains close to the equilibrium temperature necessary for agarose solation, thereby avoiding unnecessary thermal elevation and limiting the potential thermal impact on nearby neurites or somata.

This approach enables the flexible, iterative construction of neuronal networks in a single culture environment without predefined masks or molds. Furthermore, because the microstructures can be fabricated or modified post-seeding, this method offers a unique advantage over conventional microprinting and microstructure techniques, which require static designs established prior to cell cultivation.

### 2.2. Co-Culture with Glial Cells for the Enhanced Long-Term Cultivation of Neuronal Networks

**Figure 2:**
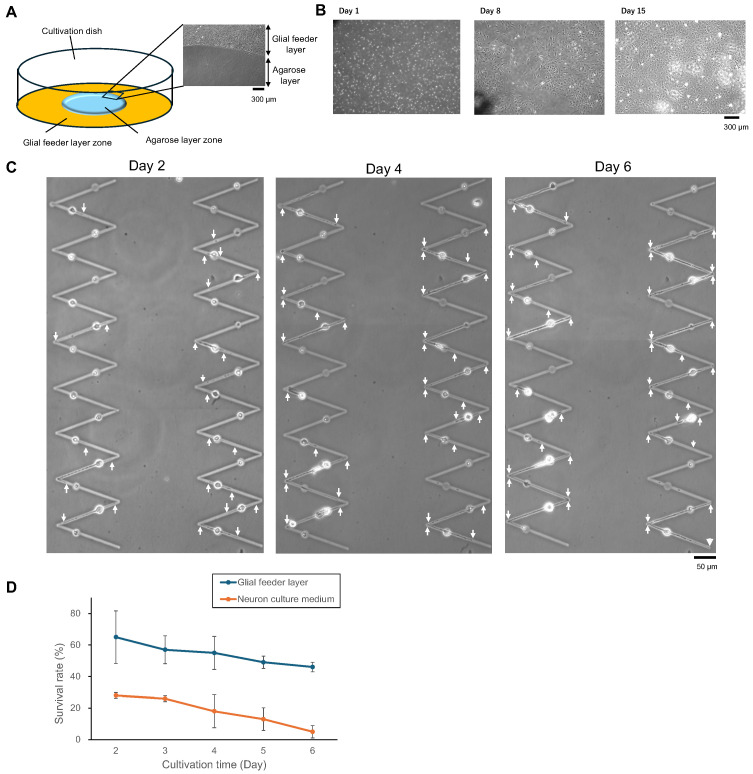
Glial co-culture enhances neuronal survival and neurite stability in long-term culture. (**A**) Experimental setup and representative micrograph showing the two-zone co-culture system. Neurons were seeded on the micropatterns fabricated in an agarose gel-coated zone adjacent to a confluent glial cell feeder layer. The glial cell feeder layer zone surrounds the agarose gel layer zone. The agarose gel layer restricts glial migration, enabling localized neurotrophic support without direct overgrowth. (**B**) Time-lapse imaging of the glial feeder zone over 15 days. Glial cells reached confluence and maintained a stable monolayer throughout the culture period. (**C**) Representative time-course images showing neurite elongation and survival of a single neuron co-cultured with glial cells. White arrowheads indicate the tips of elongated neurites, which exhibit progressive elongation from Day 2 to Day 6 after neurons were placed into the agarose microstructures. (**D**) The time-dependent survival rate of neurons in glial co-culture and neuron culture medium conditions over 6 days after neurons were placed into the agarose microstructures (mean and standard deviation (SD; Error bars), N = 6; N represents the number of cultivation trials). Neuronal survival was significantly enhanced by the presence of glial cells, demonstrating the protective and supportive effects of co-culture.

To assess the influence of glial cells on the long-term viability and structural stability of neurons, we established a co-culture system and compared it to a neuron-only condition (Figure 2A). In the glial co-culture setup, glial cells were seeded onto designated feeder zones and allowed to reach confluence in a modified NB medium containing 10% FBS. The center agarose layer acted as a physical barrier to cell migration and the process extension of glial cells, while still allowing the diffusion of soluble factors such as gliotransmitters and neurotrophic molecules. After preconditioning the glial layer in 1% FBS medium for 24–48 h, neurons were seeded on adjacent agarose gel-coated areas, spatially isolated from direct glial contact but still exposed to glial-derived soluble factors. This configuration allowed for localized neurotrophic support while minimizing glial overgrowth into neuronal zones.

The glial feeder layer maintained a stable monolayer over 15 days without significant migration onto the neuronal zone (Figure 2A inserted micrograph and Figure 2B), ensuring consistent support throughout the experiment. The time-lapse imaging of individual neurons co-cultured with glia revealed progressive neurite extension over the first 6 days after neurons were placed into the agarose microstructures (Figure 2C). White arrowheads indicate the tips of elongating neurites, which maintained clear polarity and structural integrity across the entire observation window.

We further monitored neuronal survival over time under neuron culture medium-only and glial co-culture conditions. By Day 6, neurons in the co-culture condition exhibited a markedly higher survival rate than those in the neuron-only group (Figure 2D), confirming the long-term viability benefits of glial presence. In contrast, neurons cultured in the absence of glia showed progressive detachment, neurite retraction, and cell body shrinkage beginning as early as Day 3.

These findings demonstrate that direct co-culture with glial cells offers sustained neurotrophic and structural support, resulting in enhanced neuronal adhesion, process extension, and long-term survival. This strategy thus provides a robust foundation for constructing stable neuronal networks over extended culture durations.

### 2.3. Stepwise Modification of Agarose Gel-Confinement Structure During Cultivation for Multiple Wiring

To investigate the feasibility of the dynamic, sequential wiring of neurons through in situ microstructure modification, we performed a time-resolved neurite elongation experiment that follows the conceptual fabrication flow illustrated in Figure 1B. This experiment was designed to test whether new microchannels could be fabricated across already-extended neurites without inducing damage, and whether second neurites could cross pre-existing ones under tightly controlled geometric conditions.

As shown in Figure 1B, the procedure followed a stepwise process: (a–c) First, a circular microchamber was fabricated in the agarose gel layer via localized IR laser melting. A 5 μm-wide linear microchannel was then fabricated by translating the dish under constant laser irradiation. (d) A single neuron was manually placed into the first chamber using a glass micropipette. (e–g) After cultivation began, the neuron adhered and extended a neurite through the preformed microchannel. (h–j) A second microchamber and intersecting microchannel were then fabricated. This time, the laser path intentionally passed directly over the existing neurite. (k) A second neuron was then introduced into the newly fabricated chamber. (l) The second neuron adhered and extended a neurite through the second microchannel, ultimately crossing the trajectory of the first neurite at a defined intersection.

**Figure 3:**
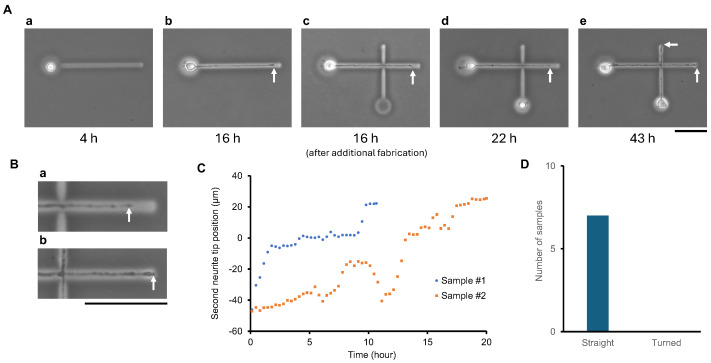
Stepwise modification of agarose gel microstructures enables sequential multi-neurite wiring and controlled neurite crossing. (**A**) Time-lapse sequence showing the dynamic fabrication and cultivation of a two-neuron circuit with intersecting neurites. (**a**) Four hours after cultivation started, the first neuron was placed into a pre-fabricated microchamber. (**b**) By 16 h, the first neurite fully elongated through the pre-fabricated initial microchannel. (**c**) After verifying full elongation, a second microchamber and a perpendicular microchannel were fabricated across the path of the first neurite. Then, the second neuron was introduced into the newly formed second chamber. Laser patterning passed directly over the existing first neurite without causing visible damage. (**d**) At 6 h after the second neuron was seeded, the second neurite initiated extension into the new microchannel. (**e**) By 43 h, the second neurite successfully crossed the first neurite at the intersection point. White arrows indicate the elongated neurite tips over time. (**B**) Enlarged time-lapse images of neurite extension following laser-based cross-channel fabrication. (**a**,**b**) High-magnification views corresponding to panels (**Ad**) and (**Ae**), respectively. (**C**) Two representative examples of second neurite elongation paths over time. The position of the second neurite tip is plotted relative to the crossing point (set as zero on the Y axis). In both cases, neurites temporarily paused near the intersection before resuming elongation and successfully crossing the first neurite. (**D**) Summary of neurite crossing behaviors. In all seven tested samples, second neurites successfully crossed the first neurite, confirming the reproducibility of the stepwise wiring strategy. Scale bars: 50 μm.

A single neuron was first introduced into a pre-fabricated microchamber connected to a linear microchannel. At 4 h post-seeding (Figure 3(Aa)), the neuron adhered and initiated neurite outgrowth. By 16 h (Figure 3(Ab)), the first neurite fully extended through the first microchannel. After confirming complete elongation, a second microchamber and intersecting microchannel were fabricated using the infrared laser melting of agarose gel (Figure 3(Ac)). Importantly, the new microchannel was directed across the path of the first neurite. Despite the laser spot crossing the existing neurite, no structural damage or morphological change was observed, confirming the minimal invasiveness and high compatibility of the fabrication process.

Following the second chamber’s formation, a second neuron was introduced. Six hours after seeding (twenty-two hours in total) (Figure 3(Ad)), the second neurite began extending through the newly formed microchannel. By 43 h (Figure 3(Ae)), the second neurite had successfully elongated through the newly fabricated channel, crossing over the first neurite without deviation or damage.

To further illustrate the continuity of neurite elongation across fabricated intersections, we included the high-magnification time-lapse images of the first neurite’s tip before and after crossing additional agarose microfabrication (Figure 3(Ba,b)). These close-up views correspond to the wide-field images shown in Figure 3(Ad,e). These close-up images reveal that the first neurite continued to elongate even after the additional laser processing. Although functional assessment was not conducted, no substantial morphological damage was observed in the elongating neurite, suggesting that laser modification does not cause overt disruption to neurite extension under the applied conditions.

The detailed time-lapse analysis of two representative examples of neurite elongations (Figure 3B) revealed a common elongation behavior: as the second neurite approached the intersection with the first neurite, growth temporarily paused upon contact. The neurite tip appeared to attach to the surface of the perpendicular first neurite. After this transient halt, elongation resumed, and the second neurite successfully crossed the first, continuing its path within the microchannel. This observation suggests that neurite–neurite contact at orthogonal crossings may cause temporary growth cone stalling but not permanent inhibition.

Consistent with this, all seven samples tested demonstrated the successful second neurite crossing of the first neurite (Figure 3C), confirming the reproducibility of the system. These results establish that our system enables real-time, multi-stage circuit construction—even including perpendicular neurite crossings—without compromising pre-existing processes or requiring the reconfiguration of the culture environment. As shown in Sample 2, even using PDL decoration, when adhesion between the growth cone and the poly-D-lysine (PDL)-coated surface is disrupted, the rapid retraction of the neurite is often observed within a short time frame. Interestingly, such retraction events are frequently followed by the accelerated re-extension of the neurite, typically up to a certain length.

**Figure 4:**
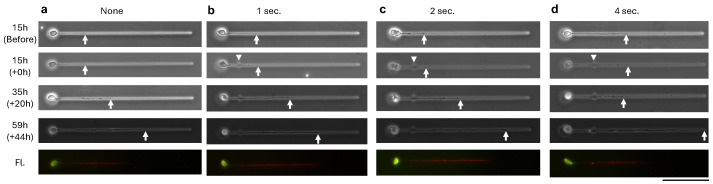
Time-lapse phase-contrast and fluorescence micrographs of neurite elongation after infrared laser irradiation at neurite contact sites. Cultures were initiated on agarose-patterned substrates, and at 15 h (15 h) post-seeding, laser irradiation was applied directly at the neurite shaft (white arrowhead) for 0 s (**a**; a non-irradiated control), 1 s (**b**), 2 s (**c**), or 4 s (**d**). Follow-up images were acquired at 35 h (+20 h post-irradiation) and 59 h (+44 h post-irradiation). White arrows indicate neurite tips. The bottom row (Fl.) shows immunofluorescence staining for MAP2 (green) and Tau1 (red) performed at 59 h. Scale bar: 100 μm.

**Figure 5:**
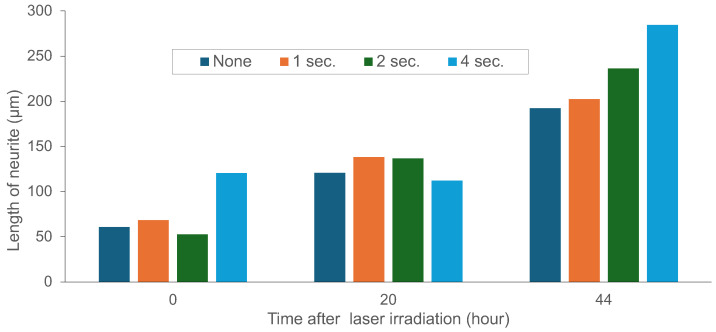
Quantitative analysis of neurite elongation following infrared laser irradiation at neurite contact sites (see Figure 4). Despite exposure durations of up to 4 s, substantially longer than the <1 s pulses used for actual agarose microfabrication (Figure 3), neurites continued to extend without interruption.

To directly assess whether infrared laser irradiation during agarose microfabrication could damage growing neurites, we conducted controlled laser exposure experiments targeting the shaft of extending neurites (Figure 4 and Figure 5). At 15 h post-seeding, neurites were exposed to a focused 1480 nm laser for 1, 2, or 4 s durations, exceeding those used during actual channel fabrication (<1 s; e.g., 0.16 W, 5 μm/ s movement), as shown in Figure 1 and Figure 3. Control neurites without irradiation were also examined (Figure 4a). Time-lapse imaging revealed that neurites under all conditions continued to elongate over the following 44 h (59 h post-seeding), with tip positions advancing beyond the irradiated regions. Immunofluorescence confirmed that the extended neurites were positive for Tau1, a marker of axonal identity, while MAP2 expression was also observed in cell bodies. These results indicate that, at least in terms of elongation, infrared laser exposure sufficient to melt agarose did not produce overt damage to the contacted neurites.

Together, these results confirm that our platform enables sequential, real-time circuit assembly, in which new neuronal pathways can be created dynamically without compromising pre-existing ones. This approach provides a powerful model for investigating neurite–neurite interaction, collision behavior, and contact-mediated pathfinding under precisely defined geometric constraints.

### 2.4. Ability and Limitation of Modifiable Agarose Gel Platforms for Crossing Multi-Neurite Wiring

In this study, we developed and demonstrated a constructive neuroengineering platform that enables the real-time modulation of neuronal wiring through the use of infrared laser-fabricated agarose gel microstructures. This system provides a new level of flexibility for guiding multi-neurite elongation and building-structured neuronal circuits with high spatiotemporal precision. Our results show that the platform supports long-term cultivation, dynamic remodeling, and intersectional neurite wiring—achievements that are difficult or impossible to realize with conventional static microfabrication methods.

A key innovation of our approach lies in the ability to fabricate and modify microchannels and cell chambers dynamically during culture, using an infrared laser system under aqueous, live-cell conditions. Unlike traditional microprinting or lithographic techniques [4], which rely on static pre-patterned substrates and cannot accommodate changes after seeding, our method enables on-demand structural modifications without compromising neuronal viability or existing neurites. This feature is especially critical for modeling dynamic neural development or for iteratively constructing complex, multistep networks, as we demonstrated through sequential neuron placement and neurite crossing.

In addition to the structural observations described in Figure 3, we further examined the potential cytotoxic effects of infrared laser irradiation on neurites during microfabrication. Our results (Figure 4 and Figure 5) demonstrate that direct laser exposure for up to 4 s, much longer than the durations used in real-time patterning, did not visibly impair neurite elongation, as neurites continued to grow past the irradiated site over the subsequent 44 h. Moreover, Tau1 immunostaining confirmed axonal marker expression in the extended processes. While these findings suggest that infrared laser modification is minimally disruptive to neurite extension, it remains unclear whether more subtle effects, such as alterations in gene expression or synaptic protein localization, may occur. Future work using additional molecular markers and electrophysiological assays will be needed to fully assess neuronal functionality after laser exposure.

This work also positions our platform as a valuable interface for studying axonal computation. As discussed by Mateus et al. [4], axons are not merely passive transmission cables but actively contribute to information processing through mechanisms such as analog–digital facilitation, plasticity, and bidirectional propagation. The ability to spatially and temporally modify axon trajectories in our system provides a basis for exploring these computational dynamics using high-density microelectrode arrays or optogenetic tools.

Furthermore, our system captures critical growth behaviors such as growth cone stalling, physical contact detection, and adaptive trajectory changes—phenomena typically attributed to the sensitivity of growth cones to guidance cues [25]. Such behaviors are foundational to both physical guidance strategies (e.g., engineered grooves and micropillars) and chemical patterning techniques (e.g., microcontact-printed adhesion molecules) [26], which traditionally rely on predefined, immobile templates. In contrast, our system enables similar or superior control while preserving temporal flexibility.

Another major finding of this study is the importance of glial support for achieving long-term neuronal viability and structural integrity. Our comparative analysis of direct glial co-culture conditions revealed that the co-culture setup consistently supported robust neurite extension and stable network maintenance for several weeks in agarose microstructures. This supports previous findings that glial cells provide both soluble trophic factors and dynamic metabolic and structural support that are essential for neuronal health over extended periods [20,27,28]. The modular feeder zone design used here ensured the spatial separation of glia and neurons while still maintaining trophic support, enabling the clear observation and manipulation of neuronal morphology.

Moreover, the stepwise neurite wiring experiments revealed the potential of this platform for constructing multi-path, directionally organized circuits, including neurite crossings in two-dimensional planes. Importantly, we showed that second neurites could cross over previously formed ones without causing damage, and even when growth cone advancement temporarily paused at intersection sites, elongation ultimately continued in all tested cases. This behavior mirrors in vivo phenomena where growth cones navigate through crowded and intersecting axonal environments, responding transiently to contact-mediated cues. Our platform thus offers a powerful tool for studying such interactions in a highly controllable in vitro setting.

An additional advantage of our dynamic agarose microfabrication system is its flexibility in constructing networks only after confirming the identity of individual cells. In practical settings, it is often difficult to definitively distinguish neurons from glial cells at the time of initial plating, particularly in primary cultures. Our system allows for a real-time observation of cell morphology and marker expression before initiating microchannel fabrication. Once a neuron is identified based on morphological or molecular criteria, it can then be selectively connected to other verified neurons using laser-guided microstructure addition. This approach is fundamentally incompatible with conventional pre-patterned platforms, where the spatial configuration must be fixed in advance and cannot be adjusted once neurite outgrowth begins. By enabling post hoc network construction based on confirmed cell identity, our method offers a uniquely adaptable strategy for constructing targeted neuronal circuits.

The overall biocompatibility of our system was indirectly supported by the sustained survival of neurons over multiple days in culture (Figure 2) and the absence of morphological abnormalities during and after laser microfabrication (Figure 3, Figure 4 and Figure 5). In addition, agarose has been widely recognized as a biocompatible substrate in neuronal culture systems [22,23,24].

While our results demonstrated preserved neurite morphology and marker expression after laser exposure, they do not exclude the possibility of subtler molecular or transcriptional alterations. In future studies, we plan to perform quantitative analyses using molecular damage markers such as HSP70, Caspase-3, and viability assays including Live/Dead staining, to more rigorously validate the biological safety and long-term biocompatibility of laser-based microfabrication.

Compared to existing scaffold-based systems, our approach offers several advantages: (1) Dynamic adaptability, enabling an iterative network design. (2) Biocompatibility, with no detectable thermal or mechanical damage to living neurons. (3) High precision, allowing neurite-level resolution in channel geometry and neuron placement. (4) Long-term stability, supported by glial co-culture and agarose gel biocompatibility.

There are, however, some limitations to note. The system currently requires manual neuron placement, which could limit scalability. Incorporating automated cell positioning or integrating optogenetic stimulation and calcium imaging could further enhance its functionality for circuit-level analysis. Additionally, while two-dimensional configurations were demonstrated here, future work may explore vertical (3D) channel architectures to more closely mimic in vivo neural tissue structures.

In conclusion, our modifiable agarose gel platform represents a versatile and powerful strategy for constructive neuroengineering, enabling the flexible and stepwise wiring of neuronal networks with real-time structural control. This system opens new possibilities for investigating neuronal connectivity, plasticity, and regenerative strategies and may serve as a foundation for future applications in disease modeling, brain-on-chip technologies, and neuromorphic engineering.

## 3. Conclusions

This study presents a novel, dynamically modifiable agarose gel-based platform for the constructive neuroengineering of long-term neuronal networks with precisely guided multi-neurite wiring. By integrating the real-time infrared laser microfabrication with glial co-culture support, we enabled spatiotemporally controlled neurite elongation, iterative circuit assembly, and even orthogonal neurite crossings, without disrupting existing cellular structures. The platform’s adaptability, biocompatibility, and long-term stability represent a significant advancement over conventional static systems, offering powerful new capabilities for building and studying complex, functional neuronal architectures in vitro. While the current study focused on morphological and structural validation, it does not include direct assessments of apoptosis, proliferation, or synaptic function. These remain important future directions. Planned follow-up studies will include molecular viability assays (e.g., HSP70, Caspase-3, Live/Dead staining) and electrophysiological recordings using multi-electrode array (MEA) systems to evaluate the functional connectivity and physiological activity of dynamically wired neuronal networks. Clarifying these aspects will further validate the platform’s utility for long-term and functional neuroengineering applications.

## 4. Materials and Methods

This study was conducted in accordance with the Act on Welfare and Management of Animals of the Ministry of the Environment, Japan. All animal experiments and protocols were approved by the Waseda University Animal Experiment Committee (permission numbers: A23-096 and A24-099) and adhered to ARRIVE 2.0 guidelines.

### 4.1. Agarose Gel Photo-Thermal Microfabrication System

To microfabricate, we used a 1480 nm infrared laser photo-thermal etching system. The system consists of three parts: a phase-contrast microscope (IX-71 with 20× phase-contrast objective lens, LCUPlanFL N, OLYMPUS, Tokyo, Japan), a motorized x–y stage (BIOS-206T, SIGMA KOKI Co., Ltd., Tokyo, Japan), and a 1480 nm focused laser irradiation module (RLM-1-1480, IPG Laser, Oxford, MA, USA). Two wavelengths of light were used simultaneously: 520 nm visible light for phase-contrast microscopy and 1480 nm laser for photothermal etching. Phase-contrast images were acquired using a charge-coupled device CCD camera (CS230, OLYMPUS, Tokyo, Japan).The dichroic mirrors and lenses in the system were chosen for their suitability for these two wavelengths.

### 4.2. Agarose Gel Microfabrication

The agarose gel microstructures on the 35 mm tissue dish (300-035, AGC Technoglass Co., Ltd., Shizuoka, Japan) were prepared as follows. First, the 35 mm dish was made hydrophilic with a plasma ion bombarder (PIB-20, Vacuum Device Inc., Ibaraki, Japan). Then, the bottom of the 35 mm dish was immersed in 100 μL of 1 mg/mL Poly-D-lysine (P0899, Sigma-Aldrich Co., LLC., Tokyo, Japan). After 15 minutes, the immersed dish was rinsed 3 times with sterilized water to remove liquid Poly-D-lysine and dried for 15 min. Next, 1 mL sterilized water was immersed in the dish, and 650 μL of the water was removed. Then, 85 μL of 3.5% low-melting point agarose gel (melting point = 65 °C; E-3126-25, BM-BIO BM Equipment Co., Ltd., Tokyo, Japan) was spread on the dish and spin-coated with a spin coater (1H-D7, MIKASA, Tokyo, Japan) at 500 rpm for 3 s and subsequently 3000 rpm for 18 s. After this, 2 mL of water was added to the dish. To lower the temperature of the agarose gel, the dish was chilled. A focused 1480 nm laser was irradiated to dissolve the agarose gel layer partially into a sol state. Micropatterns were fabricated by moving the cultivation dish using an automated x–y stage.

### 4.3. Cell Cultivation

Rat hippocampal neurons were isolated and purified from day 18 (E18) Wistar rat embryos (Tokyo Laboratory Animal Science, Tokyo, Japan) using neuron dissociation solutions involving enzymatic digestion followed by density gradient centrifugation (291-78001, FUJIFILM Wako Pure Chemical Co., Osaka, Japan). The hippocampal neurons were cultured with neuron culture medium (148-09671, FUJIFILM Wako Pure Chemical Co., Osaka, Japan) on the agarose gel-patterned poly-D-lysine-coated 35 mm dish at 37 °C under 5 % CO_2_ at saturated humidity. The neurons were placed individually into each round-shaped agarose gel-free pattern (cell chamber) one-by-one with a fire-polished fine glass pipette. All glass pipettes were pre-washed by the manufacturer using an ultrasonic cleaning process prior to use, ensuring cleanliness and minimizing contamination.

Glial cells used in this experiment were obtained from the fetal cerebral cortex of embryonic E18 Wistar rats (Tokyo Laboratory Animal Science, Tokyo, Japan). Cerebral cortices were harvested through dissection and dissociated into single cells using Trypsin-EDTA solution (Gibco, Thermo Fisher Scientific, Waltham, MA, USA). To reduce the viscosity caused by DNA released from lysed cells, DNase (4 U/μL) was added during the dissociation process. The isolated glial cells were suspended in modified Neurobasal (NB) medium containing 10% heat-inactivated fetal bovine serum (FBS; Gibco, Thermo Fisher Scientific, Waltham, MA, USA), 2% B-27 Serum-Free Supplement (50×) liquid (Thermo Fisher Scientific, Waltham, MA, USA), 100 U/mL penicillin–streptomycin (Gibco, Thermo Fisher Scientific, Waltham, MA, USA), and 1% GlutaMAX (100×) (Gibco, Thermo Fisher Scientific, Waltham, MA, USA). Approximately 3 mL of modified NB FBS medium was used to culture the cells in a 60 mm tissue culture dish. The cells were incubated at 37 °C under 5% CO_2_ until they reached sufficient confluence.

To systematically evaluate neuronal development and network formation, two culture conditions were prepared: neuron culture medium only, and glial co-culture. For the glial co-culture condition, glial cells were first seeded into designated feeder zones and cultured in modified NB medium containing 10% FBS until they reached confluence. The medium was then replaced with modified NB medium containing 1% FBS and preconditioned for 24–48 h to allow for the accumulation of neurotrophic and extracellular matrix factors prior to neuronal seeding.

To ensure optimal pH and physiological conditions, all cell cultivation— including neuronal mono-culture and glial co-culture—was conducted inside a standard CO_2_ incubator maintained at 5% CO_2_ and 37 °C. The Neurobasal-based culture media used in this study rely on sodium bicarbonate buffering, making CO_2_ supplementation essential. For the laser-based microstructure fabrication performed before neuronal seeding, the culture dish was briefly handled outside the incubator (<20 min), with no cells present. For live-cell modification, the procedure was completed within 5 min per dish at room temperature, and cultures were promptly returned to the incubator.

### 4.4. Cell Observation

Neurons on the pattern were observed using inverted optical microscopy (IX-71 with a 20× phase-contrast objective lens, LCPlanFl, OLYMPUS, Tokyo, Japan) with a charge-coupled-device (CCD) camera imaging system (1501M-GE, THORLABS, Newton, NJ, USA). Neuronal viability was operationally defined based on morphological criteria observable under phase-contrast microscopy. Specifically, a neuron was regarded as viable if it exhibited adherence to the substrate and actively extended at least one neurite. The results were evaluated as the survival rate (mean ± S.D. %).

### 4.5. Immunofluorescence Staining

The immunostaining procedure was conducted using a modified version of a method described in our previous research [18,24]. For the immunostaining of axons, Anti-Tau-1 (MAB3420, Sigma-Aldrich Co. LLC., Tokyo, Japan) was used as the primary antibody, and Goat anti-Mouse IgG2a Cross-Adsorbed Secondary Antibody, Alexa Fluor 555 (Thermo Fisher Scientific) as the secondary antibody. For the immunostaining of dendrites, Anti-MAP2 (SIGMA ALDRICH M4403, mouse monoclonal IgG1) was used as the primary antibody, and Rabbit anti-Goat IgG Cross-Adsorbed Secondary Antibody, Alexa Fluor 488 (Thermo Fisher Scientific) as the secondary antibody. The fluorescence images were recorded with a charge-coupled device (CCD) camera imaging system (1501M-GE, THORLABS, Newton, NJ, USA).

## Data Availability

The original contributions presented in this study are included in the article. Further inquiries can be directed to the corresponding author.
